# *Taxus baccata* intoxication: the sun after the electrical storm

**DOI:** 10.5935/0103-507X.20210019

**Published:** 2021

**Authors:** Alexandre Pinto, Tiago Lemos, Inês Silveira, Irene Aragão

**Affiliations:** 1 Centro Universitário do Porto EPE - Porto, Portugal.

**Keywords:** *Taxus baccata*/toxicity, Needles, Poisoning, Death, Ventricular fibrillation, Emergency medical services, *Taxus baccata*/toxicidade, Agulhas, Envenenamento, Óbito, Fibrilação ventricular, Serviços médicos de emergência

## Abstract

European yew (*Taxus baccata*) is a tree with alternate branchlets, green needles and reddish-brown bark. A high-dose ingestion of *Taxus baccata* for suicidal purposes usually results in death. The systemic toxicity is mainly cardiac. The authors describe the case of a young patient who ingested a high dose of yew needles and presented to the emergency department with a serious intoxication, which manifested as a chaotic malignant arrhythmia that was successfully treated after exhaustive supportive care.

## Abstract (pt)

O teixo europeu (*Taxus baccata*) é uma árvore com pequenos ramos alternados, agulhas verdes e casca marrom-avermelhada. A ingestão de uma alta dose de *Taxus baccata* com intenção suicida geralmente resulta em morte. A toxicidade sistêmica é principalmente cardíaca. Os autores descrevem o caso de uma jovem paciente que ingeriu uma alta dose de agulhas de teixo e foi trazida ao pronto-socorro com intoxicação grave, manifestada por arritmias malignas caóticas, tratadas com sucesso após exaustivos cuidados de suporte.

## INTRODUCTION

European yew is common as an ornamental landscaping plant, but few people are aware of its poisonous properties. All parts of a yew, except the fleshy aril, are toxic, having the potential to release taxine-derived alkaloids, taxane-derived substances and glycosides.^(1)^

A high-dose ingestion of *Taxus baccata* for suicidal purposes usually results in death.^(2)^ The taxine alkaloids block sodium and calcium channels and disrupt sodium-potassium transport, such as digitalis glycosides, which could progress to life-threatening arrhythmia.^(3)^

Symptoms initially expected after ingestion are dizziness, mydriasis, nausea, vomiting, abdominal pain, tachycardia and convulsions, followed by bradycardia, paralysis, diastolic cardiac standstill and death.^(4)^ There are no antidotes nor specific therapy.

Conventional Advanced Life Support measures should be rapidly employed to avoid progression to the asystole phase.

## CASE REPORT

A 23-year-old female student was found lying on the street and was transported to the emergency room by paramedics.

Upon admission, she was drowsy but relatively cooperative. Cardiac monitoring showed a bizarre rhythm of extreme bradycardia (30 beats/minute) with a wide regular QRS complex. Her blood pressure was 55/30mmHg, and her oxygen saturation was 99% with a Hudson mask. The National Anti-Poison Information Center was contacted after the patient showed a piece of paper with the word “yew” written on it.

A fluid bolus with normal saline was performed, together with the administration of 1mg atropine and 2g magnesium sulfate, and a 10µg/kg/minute perfusion of dopamine was initiated.

An arterial catheter and central venous line were placed. The arterial blood sample for blood gases revealed metabolic acidosis, with a pH of 7.406, sodium bicarbonate (HCO_3_) of 16.9mmol/L, lactate level of 4.9mmol/L, partial pressure of oxygen (PaO_2_) of 307mmHg and partial pressure of carbon dioxide (PaCO_2_) of 21.5mmHg.

**Figure 1:**
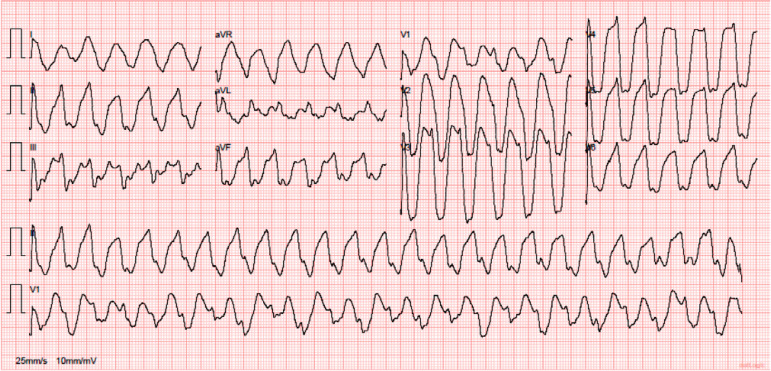
Tachycardia with a wide QRS complex.

After that, the baseline electrocardiogram (ECG) showed tachycardia at a rate of 119/minute with a wide QRS complex (0.257s) (Figure 1). The patient’s condition worsened, with a relapse of extreme bradycardia and decreased level of consciousness, and rapid sequence intubation with 20mg etomidate and 50mg of rocuronium bromide was performed.

A change in rhythm was noted; new tachycardia with a broad QRS complex. One hundred milligrams of lidocaine was administered, and the patient presented with asystole. After 2 minutes of cardiopulmonary resuscitation (CPR) and 1mg of epinephrine, the patient recovered to ventricular tachycardia (VT) with a pulse, which rapidly evolved into a new relapse of extreme bradycardia, and the patient went in asystole again after transcutaneous pacing support. The patient returned to VT with a pulse after 2 minutes of CPR and 1mg of epinephrine.

A decision regarding synchronized electrical cardioversion was made. After a 200J synchronized shock, the patient degenerated into ventricular fibrillation and CPR was restarted.

The return of spontaneous circulation was reached after 4 minutes of CPR and two defibrillations (defibrillation = 200J biphasic electric shock).

The vasopressor support was changed to norepinephrine (1.9µg/kg/minute), 10mL of calcium gluconate 10% solution and 10 vials of digoxin-specific antibody fragments were administered, and an infusion of amiodarone (1,200mg/24 hours) was started.

The echocardiogram documented normal-sized chambers and normal systolic and diastolic biventricular function, without evidence of valvopathies.

**Figure 2:**
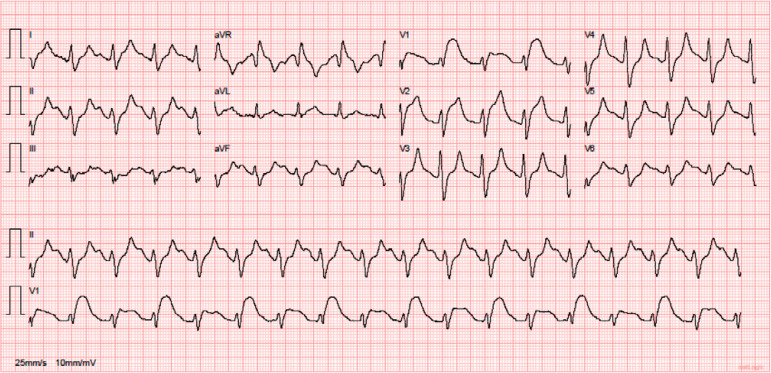
Progressive rhythm organization to sinus rhythm.

After 4 hours of exhaustive Advanced Life Support, we verified a progressive rhythm organization to sinus rhythm (ECG, Figure 2). The dose of norepinephrine was reduced to 0.1µg/kg/minute.

The patient was transferred to the intensive care unit (ICU). The laboratory tests upon ICU admission showed changes in renal and hepatic function tests, reflecting acute kidney injury and ischemic hepatitis, respectively.

The patient was extubated after 24 hours of invasive mechanical ventilation. No neurological damage was objectified, and the patient was discharged to the internal medicine ward on day 2.

The clinical condition of the patient improved. She was observed and medicated by a psychiatrist. She was discharged home on day 7.

## DISCUSSION

*Taxus baccata* is a moderately-sized ornamental evergreen conifer tree. It is widely used in landscaping and is considered an ideal plant for hedging due to its relatively slow growth and tolerance of pruning.^(1)^ In the hospital setting, *Taxus baccata* is the source of paclitaxel, a chemotherapeutic agent used in the treatment of lung cancer.

All parts of a yew, except the fleshy aril surrounding the seed, are toxic. They contain a complex mixture of compounds, including phenolic constituents (e.g., 3,5-dimethoxyphenol), nonalkaloid diterpenoids (e.g., 10-deactetylbaccatin III), alkaloid diterpenoids (e.g., taxine B, taxine A), flavonoids (e.g., myricetin) and bioflavonoids (e.g., bilobetin).^(5)^ The lethal dose for an adult is reported to be 50g of yew needles. Estimating that 1g of yew needles contains approximately 5mg of taxines, the minimal toxic dose for humans is calculated to be 3.0 - 6.5mg taxines/kg.^1^

The time from ingesting a lethal dose to death is usually 2 to 5 hours, with symptoms occurring from 30 minutes to 1 hour following ingestion.^(6)^

The major compounds of the alkaloid fraction are taxine A and taxine B.^(5)^ The latter represents 30% of the total alkaloid fraction extracted from *T. baccata* and is more potent than other taxine alkaloids, therefore having more cardiac toxicity. Taxine A represents only 1.8% of the alkaloid fraction.^(3)^

A pharmacological investigation of taxine alkaloids revealed that the hypotension induced by taxines is not mediated via the sympathetic or parasympathetic nervous system, but rather, by a direct action on the myocardium and vascular smooth muscle.^(1)^

Taxines, particularly taxine B, are cardiac myocyte calcium and sodium channel antagonists that inhibit calcium and sodium channels in the same manner as drugs such as verapamil, although taxines are more potent and cardioselective.^(3)^ This cardiotoxicity is manifested by negative inotropism and an atrioventricular conduction delay that increases the electrocardiographic QRS complex duration; the P wave can also be absent, as seen in the first ECG. The QRS width can be explained by the degree of inhibition of the fast cardiac sodium channels during phase 1 of the action potential.

Like other calcium channel antagonists, taxines also suppress vascular smooth muscle contraction and can produce marked arterial vasodilation-mediated hypotension.^(7)^ This is the reason why the patient was in vasoplegic shock at admission.

Our patient initially presented with a disorganized, wide QRS complex bradycardic rhythm, which usually precedes electromechanical dissociation, subsequent asystole and death.^(8)^ In the early stage of poisoning, the ECG may show multiple extrasystoles, followed by persistent VT.

Since there is no specific antidote or evidence-based anti-arrhythmic therapy, rapid supportive care is crucial to avoid progression to death.^(9)^ In our case, despite the exhaustive supportive treatment carried out, we saw recurring malignant ventricular arrhythmias. Due to this, we used intravenous lidocaine, but it was ineffective.

Although we did not consider the possibility of provisional invasive pacing, we think it should be considered in similar cases due to the possibility of having to perform overdrive pacing during VT.

After the two episodes of ventricular fibrillation, ten vials of digoxin-specific antibody fragments were administered. After this, we verified a progressive rhythm organization to sinus rhythm, possibly due to the absorption of taxine by the Fab fragments, thereby eliminating the free, unbound portion of the alkaloid.^(9,10)^

In the literature, there are several case reports that describe the successful use of extracorporeal membrane oxygenation to support the circulation of patients with refractory ventricular arrhythmias and consequent cardiogenic shock, thus allowing time for toxin metabolism, rhythm stabilization and myocardial recovery.^(11)^

Continuous renal replacement therapy or hemodialysis was not considered since they are unlikely to be effective due to the large volume of distribution and the high protein binding of taxine A.^(12)^

## CONCLUSION

Yew (*Taxus baccata*) is an evergreen commonly used for ornamental landscaping. Taxines are poisonous constituents in yew plants that block sodium and calcium channels in the heart, leading to life-threatening cardiotoxicity (atrioventricular block, ventricular tachycardia and refractory ventricular fibrillation). Patients who ingest a lethal dose frequently die, despite resuscitation efforts. Serious poisoning occurs in the setting of suicidal ingestion. In patients who present with signs of high toxicity, the clinician should rapidly perform an initial assessment of clinical findings and support the airway, breathing and circulation as needed.

Since there is no known antidote, management of yew intoxication is essentially supportive, requiring intensive care management with vasopressor support, invasive mechanical ventilation, eventual temporary cardiac pacing, and a temporary period of extracorporeal membrane oxygenation. Treatment with calcium infusions and the administration of digoxin-specific antibody fragments may play an important role in the management of this cardiotoxic plant.

## References

[1] Wilson CR, Sauer J, Hooser SB (2001). Taxines: a review of the mechanism and toxicity of yew (Taxus spp.) alkaloids. Toxicon.

[2] Reijnen G, Bethlehem C, van Remmen JM, Smit HJ, van Luin M, Reijnders UJ (2017). Post-mortem findings in 22 fatal Taxus baccata intoxications and a possible solution to its detection. J Forensic Leg Med.

[3] Jambeih RA, Shaheen WH, Li VY, Shaheen MH (2012). ST-segment elevation and ventricular tachycardia after ingestion of a common ornamental plant-a case report. Indian Heart J.

[4] Diaz JH (2016). Poisoning by herbs and plants: rapid toxidromic classification and diagnosis. Wilderness Environ Med.

[5] Grobosch T, Schwarze B, Stoecklein D, Binscheck T (2012). Fatal poisoning with Taxus baccata: quantification of paclitaxel (taxol A), 10-deacetyltaxol, baccatin III, 10-deacetylbaccatin III, cephalomannine (taxol B), and 3,5-dimethoxyphenol in body fluids by liquid chromatography-tandem mass spectrometry. J Anal Toxicol.

[6] Piskac O, Stríbrný J, Rakovcová H, Malý M (2015). Cardiotoxicity of yew. Cor Vasa.

[7] Soumagne N, Chauvet S, Chatellier D, Robert R, Charrière JM, Menu P (2011). Treatment of yew leaf intoxication with extracorporeal circulation. Am J Emerg Med.

[8] Grobosch T, Schwarze B, Felgenhauer N, Riesselmann B, Roscher S, Binscheck T (2013). Eight cases of fatal and non-fatal poisoning with Taxus baccata. Forensic Sci Int.

[9] Willaert W, Claessens P, Vankelecom B, Vanderheyden M (2002). Intoxication with taxus baccata: cardiac arrhythmias following yew leaves ingestion. Pacing Clin Electrophysiol.

[10] Cummins RO, Haulman J, Quan L, Graves JR, Peterson D, Horan S (1990). Near-fatal yew berry intoxication treated with external cardiac pacing and digoxin-specific FAB antibody fragments. Ann Emerg Med.

[11] Baum C, Bohnen S, Sill B, Philipp S, Damerow H, Kluge S (2015). Prolonged resuscitation and cardiogenic shock after intoxication with European yew (Taxus baccata): complete recovery after intermittent mechanical circulatory support. Int J Cardiol.

[12] Dahlqvist M, Venzin R, Konig S, Faber K, Weinmann W, Terbeck S (2012). Haemodialysis in Taxus baccata poisoning: a case report. QJM.

